# Characterization of KIR ^+^ NK cell subsets with a monoclonal antibody selectively recognizing KIR2DL1 and blocking the specific interaction with HLA‐C

**DOI:** 10.1111/tan.14640

**Published:** 2022-05-15

**Authors:** Raffaella Meazza, Michela Falco, Paolo Canevali, Fabrizio Loiacono, Natalia Colomar‐Carando, Aura Muntasell, Anna Rea, Maria Cristina Mingari, Franco Locatelli, Lorenzo Moretta, Miguel Lopez‐Botet, Daniela Pende

**Affiliations:** ^1^ Laboratory of Immunology IRCCS Ospedale Policlinico San Martino Genoa Italy; ^2^ Laboratory of Clinical and Experimental Immunology IRCCS Istituto Giannina Gaslini Genoa Italy; ^3^ Department of Experimental Medicine University of Genoa Genoa Italy; ^4^ Immunity and Infection Laboratory Hospital del Mar Medical Research Institute (IMIM) Barcelona Spain; ^5^ Department of Cell Biology, Physiology and Immunology Universitat Autònoma de Barcelona Bellaterra Spain; ^6^ Department of Medicine and Life Sciences Universitat Pompeu Fabra Barcelona Spain; ^7^ Department of Hematology/Oncology and Cell and Gene Therapy IRCCS Ospedale Pediatrico Bambino Gesù Rome Italy; ^8^ Department of Gynecology/Obstetrics and Pediatrics Sapienza University Rome Italy; ^9^ Tumor Immunology Unit IRCCS Ospedale Pediatrico Bambino Gesù Rome Italy

**Keywords:** HLA‐C, killer immunoglobulin‐like receptors, KIR/KIR‐ligand interaction, monoclonal antibodies, natural killer cells

## Abstract

The phenotypic identification of different NK cell subsets allows more in‐depth characterization of KIR repertoire and function, which are of potential interest in KIR and disease association studies. *KIR* genes are highly polymorphic, but a great homology exists among the various sequences and few monoclonal antibodies (mAbs) specifically recognize a single KIR. This is the case of HP‐DM1 which was demonstrated by analysis of cell transfectants and epitope mapping to be exclusively KIR2DL1‐specific, covering all allotypes identified to date, except for KIR2DL1*022 and *020, and also to react with KIR2DS1*013. Here, we compared in immunofluorescence analyses the staining of HP‐DM1 with other available mAbs to precisely identify KIR2DL1^+^ NK cells in potential donors for αβT/B‐depleted haplo‐HSCT, with known *KIR* genotype. HP‐DM1 mAb was used in combination with EB6 or 11PB6 (anti‐KIR2DL1/S1 and anti‐KIR2DL3*005), 143211 (anti‐KIR2DL1/S5), and HP‐MA4 (anti‐KIR2DL1/S1/S3/S5) mAbs, allowing the accurate identification of different KIR^+^ NK cell subsets. These phenotypic evaluations appeared useful to dissect the expression pattern of various KIR2D in NK cells from KIR2DL3*005 ^+^ individuals, particularly if *KIR2DS1* is present. HP‐DM1 mAb remarkably refined NK cell phenotyping of donors carrying *KIR2DS5*, either in the centromeric or telomeric region. Functional assays with KIR2DL1^+^/S1^+^/S5^+^ NK cells confirmed that only HP‐DM1 exclusively reacts with KIR2DL1. Finally, we demonstrated that HP‐DM1 mAb blocked KIR2DL1 recognition of C2^+^ HLA‐C. Altogether, the data support that HP‐DM1 is a unique reagent valuable for characterizing KIR^+^ NK cell subsets.

## 1 INTRODUCTION

Natural Killer (NK) cells are considered cytotoxic components of innate lymphoid cells (ILCs) that provide the first line of defense against viruses and tumors in peripheral blood and tissues.^1^, ^2^ In uterine mucosa, tissue‐resident NK cells and ILCs exert an important role in regulating normal placental development.^3^, ^4^ NK cells keep in check the health of neighboring cells through an array of germline‐encoded receptors, upon engagement with specific ligands.^5^ The balance between activating and inhibitory signals transmitted by these receptors finely regulates NK cell function.^1^ Major NK cell receptors are KIR, a family of transmembrane proteins characterized by 2 or 3 Ig‐like extracellular domains (KIR2D or KIR3D), which include both inhibitory (iKIR) and activating receptors (aKIR).^6^, ^7^, ^8^, ^9^ While iKIR have a “long” (L) cytoplasmic tail containing ITIMs (i.e., KIR2DL and KIR3DL), aKIR have a “short” (S) cytoplasmic tail and transduce the activating signal through KARAP/DAP12 adaptor molecule. The most relevant iKIR recognize epitopes shared by distinct groups of HLA‐A, ‐B, or ‐C allotypes.^9^ The KIR family consists of four distinct lineages, which differ in structural characteristics and specific HLA binding capacity. Lineage III includes all KIR recognizing HLA‐C.^10^ Two epitopes are defined by a dimorphism at position 80 of HLA‐C, where asparagine (N80) and lysine (K80) identify the C1 and C2 groups of KIR ligands (KIR‐L), respectively.^8^, ^11^ KIR2DL1 through methionine 44 (M44) stringently recognizes C2, whereas KIR2DL2/L3 through K44 mainly recognize C1 and with low affinity C2.^12^, ^13^, ^14^, ^15^ Notably, KIR2DL1*022 represents an exception being characterized by K44 and displaying C1 recognition.^16^ For the sake of brevity, hereafter “KIR” acronym will be omitted from genes and proteins. Lineage III also includes five aKIR (*2DS1*, *2DS2*, *2DS3*, *2DS4*, and *2DS5*). The activating receptor 2DS1, sharing M44 with 2DL1, has the same specificity for C2 epitope, though with lower affinity,^17^, ^18^ whereas the ligands of the other KIR2DS are incompletely characterized.^9^, ^19^, ^20^, ^21^, ^22^

*KIR* genes are inherited as haplotypes, comprising centromeric (Cen) and telomeric (Tel) regions, separated by a recombination site. These regions are bordered by conserved framework genes (i.e., *3DL3*, *3DP1*, *2DL4*, and *3DL2*). Based on the variety of number and type of the *KIR* present, different centromeric (i.e., Cen‐A, Cen‐B1, and Cen‐B2) and telomeric (i.e., Tel‐A and Tel‐B) regions have been identified.^23^, ^24^, ^25^ Two groups of *KIR* haplotypes have been defined.^26^, ^27^ *KIR A* haplotypes are composed by Cen‐A and Tel‐A, and have a fixed and limited number of genes. They include genes encoding for iKIR recognizing HLA‐C (*2DL1*, *2DL3*), HLA‐B (*3DL1*), HLA‐A (*3DL2*), and carry only one aKIR (*2DS4*) that, in European populations, often codes for a truncated/no‐functional receptor.^28^ Differently, *KIR B* haplotypes (i.e., Cen‐A/Tel‐B, Cen‐B/Tel‐A, and Cen‐B/Tel‐B) are characterized by a great gene content diversity and by various genes encoding aKIR.^8^ Restricted to *KIR B* haplotypes are the following genes: *2DS2*, *2DL2*, and *2DL5B* located in Cen‐B, *3DS1*, *2DL5A*, and *2DS1* located in Tel‐B, while *2DS3* and *2DS5* can be present in both Cen‐B and Tel‐B. Regarding *2DS5*, ethnic differences have been observed. In populations of European origin, there is a single *2DS5* allele (i.e., 2DS5*002), which is in strong linkage disequilibrium with *2DS1* and located in Tel‐B region. In Africans and African Americans, *2DS5* can be found in both Cen‐B and Tel‐B regions, and is polymorphic. In particular, among the Cen‐B *2DS5* alleles, 2DS5*006 is highly represented in sub‐Saharan Africans and encodes for a receptor that recognizes C2.^29^ Studying pre‐eclampsia in pregnant women, when the fetus carries a C2 epitope, maternal *KIR AA* genotypes are risk factors, whereas the Tel‐B *2DS1* in Europeans and the Cen‐B 2DS5*006 in Africans are protective.^30^

Circulating NK cells of different donors show extremely variegated KIR repertoires,^31^ primarily because of the high polymorphism of *KIR* and *HLA* class I genotypes.^8^ Importantly, KIR expression primarily results from stochastic events during NK cell differentiation but is also influenced by interaction with self‐HLA class I molecules, following an education process.^32^, ^33^, ^34^, ^35^ Besides genetic factors, environmental stimuli (e.g., pathogen exposure) may also contribute to the diversity.^31^, ^36^, ^37^

The combined KIR genotype and phenotype analysis can be informative to precisely evaluate the actual frequency of specific NK cell subsets with potential clinical interest.^38^, ^39^ A relevant application aims to select suitable donors for haploidentical hematopoietic stem cell transplantation (haplo‐HSCT) in leukemia patients. In the case of KIR/KIR‐L mismatch in graft versus host direction, the size of the alloreactive NK cell subset identified in potential alternative donors can be compared.^14^, ^40^ In addition, KIR phenotyping can help to distinguish iKIR from aKIR, but the mAb specificity and combined staining strategies should be precisely defined. Indeed, we previously demonstrated that EB6B (hereafter called EB6) and 11PB6 mAb recognize not only 2DL1/S1 but also the 2DL3*005 allotype, which shares E35 and R50, two residues involved in these mAb epitopes.^41^ There is a great homology among the various KIR aminoacidic sequences, and very few anti‐KIR mAb produced so far specifically recognize only one KIR. Examples are anti‐KIR3DL1 DX9 mAb, anti‐KIR2DS4 FES172 mAb, and anti‐KIR2DL3 (except *005 and *015 allotypes) ECM‐41 mAb.^42^, ^43^, ^44^ In this article we characterized the usefulness of a unique mAb (HP‐DM1), which exclusively reacts with KIR2DL1.^45^ The use of HP‐DM1 mAb in combination with other selected anti‐KIR2D mAbs allowed the identification of distinct KIR^+^ subsets. Moreover, functional assays showed that HP‐DM1 specifically blocked the interaction of 2DL1 with HLA‐C carrying C2 epitope.

## 2 METHODS AND MATERIALS

### 2.1 Donors

All individuals described in this article are healthy potential donors for αβT/B‐depleted haplo‐HSCT (NCT01810120) analyzed for *KIR* gene profile and NK cell receptor phenotype.^40^ This study was approved by the Ethical Committee of IRCCS Ospedale Pediatrico Bambino Gesù (OPBG, Rome, Italy), Prot. No. 424/2011. Written informed consent was obtained from all donors in accordance with the Helsinki declaration.

### 2.2 *KIR* gene profile and KIR‐ligand analyses

DNA of the tested samples was extracted using QIAamp DNA Blood Mini kit (QIAGEN, GmbH, Germany). The *KIR* gene profiles were performed using Olerup SSP‐PCR (sequence‐specific primer‐PCR) KIR genotyping kit (CareDx, Stockholm, Sweden) following the manufacturer's instruction. KIR‐L were evaluated analyzing high‐resolution *HLA* class I typing with KIR‐ligand calculator program (http://www.ebi.ac.uk/ipd/kir/ligand.html).^40^ In case of low‐resolution *HLA* class I typing, analysis of KIR‐L was also performed by SSP‐PCR using KIR HLA ligand kit (CareDx). *KIR* genotypes and KIR‐L of the individuals described in this study are reported in Figure S1.

### 2.3 Isolation and culture of NK cells

Peripheral blood mononuclear cells (PBMC) were isolated by Ficoll gradient centrifugation from heparinized blood of healthy donors. NK cells, purified using the RosetteSep method (StemCell Technologies, Vancouver, BC), were cultured on irradiated feeder cells in the presence of 2 μg/ml phytohemagglutinin (Sigma‐Aldrich, Irvine, UK) and 600 IU/ml rIL‐2 (Proleukin, Chiron Corp., Emeryville, USA) to obtain proliferation and expansion of activated polyclonal NK cells. NK cell clones have been obtained culturing upon limiting dilution either purified NK cells or CD3^−^ GL‐183^−^ 11PB6^−^ HP‐MA4^+^ cells sorted from PBMC to enrich in 2DS5^+^/other KIR2D^−^ clones.

### 2.4 Monoclonal antibodies and cytofluorimetric analysis

All anti‐KIR mAb used in this study are described with the updated specificity and the fluorochrome conjugation in Table 1. The anti‐CD3‐BV510 (UCHT1, IgG1) and the anti‐CD56‐BV421 (NCAM16.2, IgG2b) were provided by BD Biosciences (San José, CA). In multi‐color fluorescence analyses using un‐conjugated HP‐DM1 mAb, NK cells were first incubated with HP‐DM1 followed by anti‐IgG1‐PE (Southern Biotechnology, Birmingham, AL), washed twice, and then incubated with the other fluorochrome‐labeled mAb. More recently, HP‐DM1‐PE (Biolegend, San Diego, CA) has been available, allowing co‐incubation with other anti‐KIR‐APC mAb. The HP‐DM1‐PE/EB6‐PC7/HP‐MA4‐APC and HP‐DM1‐PE/EB6‐APC/CH‐L‐FITC combinations were also used. Importantly, in co‐stainings including EB6 and HP‐MA4 mAbs, EB6 was always added 10 min before the other mAb(s). Flow cytometric analysis of resting NK cells (gating on CD3^−^CD56^+^ cells of PBMC) or polyclonal activated NK cells were performed on either MACSQuant Analyzer (Miltenyi Biotec GmbH, Bergisch Gladbach, Germany) or Gallios flow‐cytometer (Beckman Coulter, Brea, CA). Data were analyzed using FlowJo Version 10.7 (TreeStar, Ashland, OR).

**TABLE 1:** Anti‐KIR monoclonal antibodies used in this study, with updated specificity

Clone	Specificity	Isotype	Fluorochrome	Vendor/Reference
HP‐DM1	KIR2DL1	IgG1	Un‐conjugated, PE	Biolegend (San Diego, CA), reference [45]
143211	KIR2DL1/S5	IgG1	APC	R&D Systems (Minneapolis, MN)
EB6B	KIR2DL1/S1, KIR2DL3*005^a^	IgG1	APC, PC7, PE	Beckman Coulter (Brea, CA)
11PB6	KIR2DL1/S1, KIR2DL3*005^a^	IgG1	Un‐conjugated, FITC	Miltenyi Biotec GmbH (Bergisch Gladbach, Germany)
HP‐MA4	KIR2DL1/S1/S3/S5	IgG2b	APC	Biolegend
GL‐183	KIR2DL2/L3/S2^b^	IgG1	PE	Beckman Coulter
CH‐L	KIR2DL2/L3/S2^b^	IgG2b	FITC	BD Biosciences (San José, CA)
ECM‐41	KIR2DL3 (not E35 allotypes)	IgM	Un‐conjugated	Reference [43]
Z27	KIR3DL1/S1	IgG1	PE	Beckman Coulter
DX9	KIR3DL1	IgG1	PE‐Vio770	Miltenyi Biotec

^a^ EB6B and 11PB6 mAb also recognize KIR2DL3 allotypes characterized by E35 and R50, including KIR2DL3*005.

^b^ GL‐183 and CH‐L mAbs also recognize KIR2DL3 allotypes characterized by E35, including KIR2DL3*005.

### 2.5 Cytotoxicity assay

Reverse antibody dependent cellular cytotoxicity (R‐ADCC) against FcγR^+^ P815 target cells was performed either in the absence or presence of the indicated mAbs (0.5 μg/ml) using polyclonal activated NK cells as effectors (E:T ratio 4:1) in a 4 h ^51^Cr‐release assays. In R‐ADCC, killing is enhanced when the mAb is reacting with an NK activating receptor, while it is decreased by an anti‐inhibitory receptor mAb.^14^ In masking experiments using C1R target cells, mAbs were used at a final concentration of 20 μg/ml.

### 2.6 Analysis of *KIR2DL1*, *2DS1*, and *2DS5* transcripts

Total RNA was extracted from NK cell bulk population or clone derived from donor TB17B using RNeasy micro kit (Qiagen), according to the manufacturer's instructions. The cDNA synthesis was performed on ~1 μg RNA using oligo(dT) oligo nucleotides. The presence of *2DL1*, *2DS1*, and *2DS5* transcripts was analyzed using the sets of primers Fcg622 5′‐CCATCAGTCGCATGACG, Ra957 5′‐CCACTCGTATGGAGAGTCAT, Ra872 5′‐AATGTTCCGTTGACCTTGGT; Fr621 5′‐TCTCCATCAGTCGCATGAR, Ra899 5′‐AGGGCCCAGAGGAAAGTT; Fc551 5′‐AGAGAGGGGACGTTTAACC, R939 5′‐GGAAAGAGCCGAAGCACT, respectively.^46^

The primers used to amplify complete *2DS5* transcripts were 2DS5 ORF up: 5′‐CATGTYRCTCATGGTCATC and C: 5′‐AAAACACAGTGATCCAATTA. PCR was performed for 30 cycles: 30 s at 95°C, 30 s at 60°C, and 30 s at 72°C; the amplification product was cloned into pcDNA3.1/V5/His TOPO vector using the Eukaryotic TOPO TA Cloning kit (Invitrogen, Carlsbad, CA). DNA sequencing was performed using d‐Rhodamine Terminator Cycle Sequencing kit and a 3100 ABI automatic sequencer (PerkinElmer, Wellesley, MA).

### 2.7 Detection of 2DL3*004, *005, *010, and *036 alleles

To identify donors characterized by at least one 2DL3*004, *005, *010, or *036 alleles, genomic DNA of *2DL3* positive donors was tested using the set of primers 2DL3*005 for: 5′‐CAGAAAACCTTCCCTCCG and 2DL2/L3 rev: 5′‐TGGGCCCTGCAGAGAA. These four *2DL3* alleles code for allotypes characterized by E35, and therefore not recognized by ECM‐41 mAb. PCR was performed for 30 cycles: 30 s at 95°C, 30 s at 60°C, and 1 min at 72°C. All PCR reactions also included a set of primers amplifying a conserved fragment of *DRΑ* gene (internal control), to avoid false negative results.^47^ In this article these four *2DL3* alleles have been indicated as 2DL3*005. The detection of 2DL3*005 allele is shown in Figure S2. Additional *2DL3* alleles coding for allotypes characterized by E35 are 2DL3*014, *015, *017, *018, *033, and *035. In all *2DL3* ^+^ donors analyzed in this study, when an unexpected GL‐183^+^ ECM‐41^−^ NK cell subset was present (as in samples analyzed in Figure S3), this PCR analysis always revealed the presence of 2DL3*005.

### 2.8 Statistical analysis

Statistical analyses were performed using Graphpad software Version 6.0. The utilized test was Ordinary one‐way ANOVA for repeated measures followed by Bonferroni multiple comparison test. Not significant (n.s.); ****p < 0.0001; ***p < 0.001; **p < 0.01; and *p < 0.05.

## 3 RESULTS

### 3.1 HP‐DM1 mAb recognizes KIR2DL1 on NK cells

**FIGURE 1:**
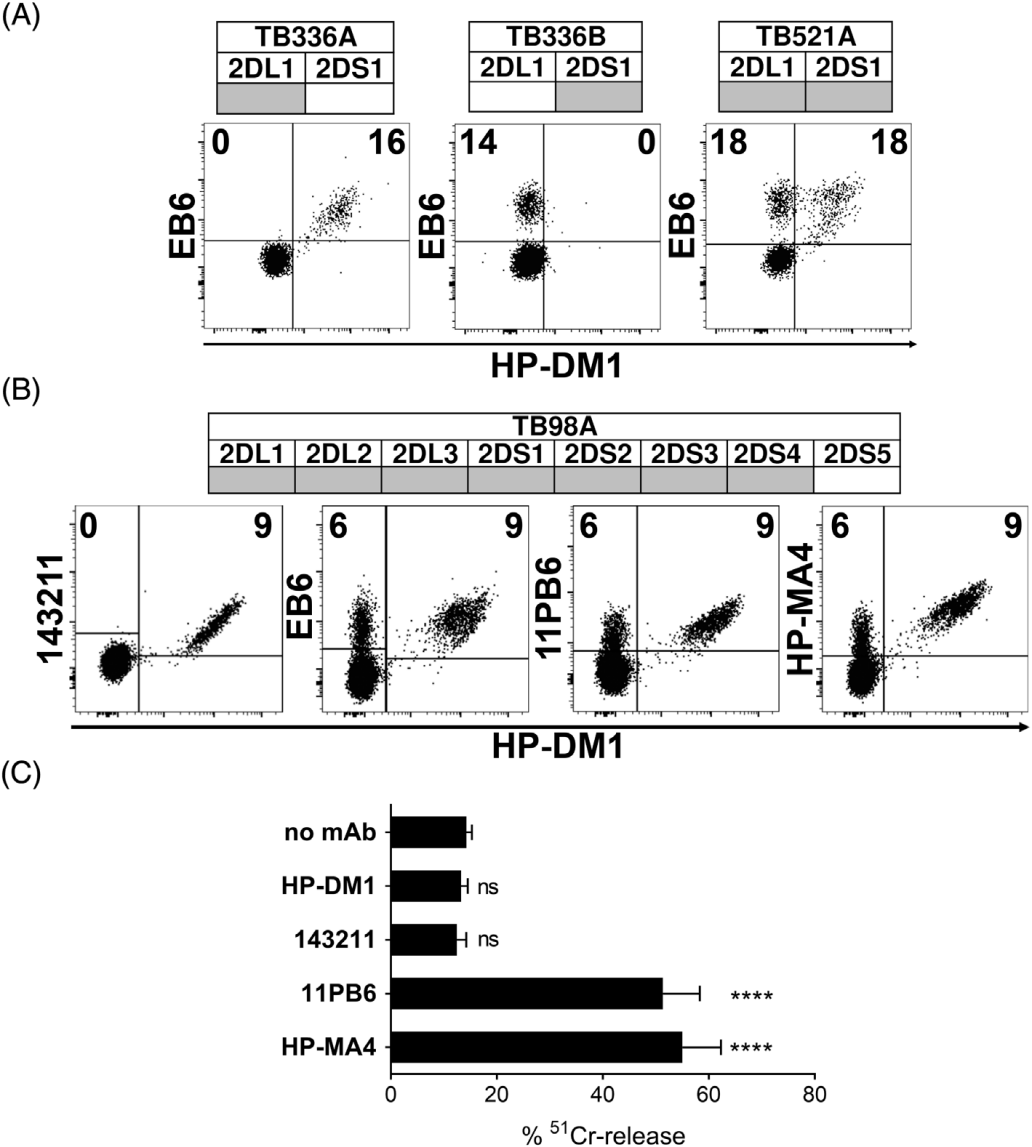
HP‐DM1 mAb selectively recognizes 2DL1 on NK cells. (A) Resting NK cells derived from three donors (TB336A, TB336B, and TB521A) characterized by different *2DL1* and *2DS1* gene profiles (positive and negative *KIR* genes are reported as gray and white boxes, respectively) were stained with HP‐DM1‐PE in combination with EB6‐APC. (B) Activated NK cells derived from a donor (TB98A), characterized by all lineage III *KIR2D* genes except *2DS5*, were stained with HP‐DM1 mAb followed by anti‐IgG1‐PE and, subsequently, with either 143211‐FITC, EB6‐APC, 11PB6‐FITC or HP‐MA4‐APC mAb. Percentages of positive cells are indicated. (C) In R‐ADCC assay, ^51^Cr‐labeled P815 were incubated with NK cells derived from different *2DL1* ^+^ */S1* ^+^ donors, including the representative donor described in (B), in the absence or presence of the indicated mAb. The *KIR* genotype and KIR‐L of these donors are reported in Figure S1. Data, obtained by nine independent experiments, are shown as mean + SEM. E:T ratio was 4:1. ****p < 0.0001

Based on the knowledge of the HP‐DM1 mAb reactivity on HEK‐293T cells transiently transfected with plasmids coding for different lineage III KIR2D,^45^ we analyzed its binding to resting NK cells derived from donors with known *KIR* gene repertoires (Figure 1 and Figure S1). HP‐DM1/EB6 double staining of NK cells derived from *2DL1* ^+^ */S1* ^−^, *2DL1* ^−^ */S1* ^+^, or *2DL1* ^+^ */S1* ^+^ donors confirmed the unique 2DL1 specificity of HP‐DM1 mAb in contrast to the ability of EB6 mAb to recognize both 2DL1 and 2DS1 (Figure 1A). Next, we analyzed polyclonal NK cell populations derived from *2DL1* ^+^ */S1* ^+^ */S5* ^−^ donors by flow‐cytometry (a representative case is shown in Figure 1B) and by R‐ADCC assay (Figure 1C), comparing HP‐DM1 with 143211, EB6, 11PB6, and HP‐MA4 mAb. HP‐DM1 and 143211 mAb co‐stained the 2DL1^+^ subset, while 11PB6 or EB6 (sharing the same KIR specificity, see Table 1) and HP‐MA4 mAb also recognized 2DS1^+^ cells and, differently from HP‐DM1 and 143211, triggered activation in R‐ADCC. In *2DL1* ^+^ */S1* ^+^ donors, HP‐DM1 discriminated in the EB6^+^, 11PB6^+^, or HP‐MA4^+^ populations two subsets according to their staining intensity, most likely corresponding to KIR2DL1^+^/S1^−^ (dim) and KIR2DL1^+^/S1^+^ (bright) cells consistent with a previous report.^48^

Taken together, these data indicated that HP‐DM1 mAb exclusively stains 2DL1 on NK cells.

### 3.2 Use of HP‐DM1 mAb for NK cell phenotyping of 2DL3*005 ^+^ donors

**FIGURE 2:**
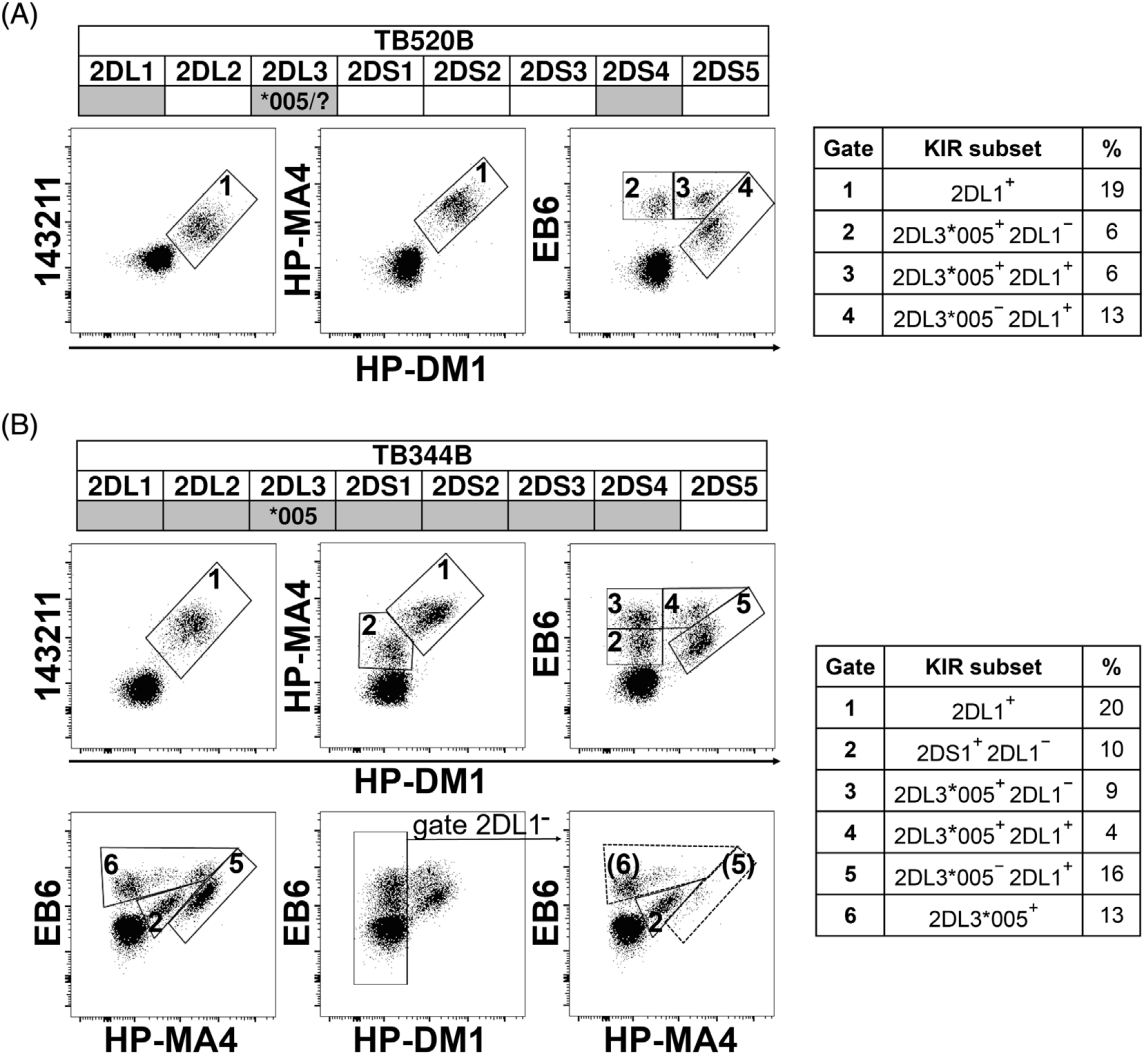
HP‐DM1 mAb allows to discriminate 2DL1^+^ from other KIR2D^+^ NK cells in 2DL3*005 ^+^ donors. Immunofluorescence analysis was performed on resting NK cells derived from two different 2DL3*005 ^+^ donors, TB520B donor (A), and TB344B donor (B). Lineage III *KIR2D* gene profiles are shown, positive and negative genes are identified as gray and white boxes, respectively. The whole *KIR* genotype and KIR‐L of TB520B and TB344B donors are reported in Figure S1. HP‐DM1‐PE was used in combination with either 143211‐APC, HP‐MA4‐APC, or EB6‐APC. Different NK cell subsets are identified by gates, which are numbered and defined for their KIR composition and percentage of NK cells included (table on the right side). In HP‐DM1‐PE/EB6‐PC7/HP‐MA4‐APC combined staining (B, lower panels), the comparison of HP‐MA4/EB6 dot plots before and after HP‐DM1^+^ cell exclusion allows to detect the localization of 2DL1^+^ cells within the different populations: the whole gate no. 5 and right part of gate no. 6 (indicated within brackets after HP‐DM1^+^ cell exclusion)

Since HP‐DM1 can selectively recognize 2DL1 allotypes (except 2DL1*022 and 2DL1*020) as demonstrated by epitope mapping,^45^ the inclusion of HP‐DM1 mAb in the panel of anti‐KIR2D reagents could contribute to more precisely define NK cell subsets. To approach this issue, we tested HP‐DM1 staining on NK cells from 2DL3*005 ^+^ donors because this allele encodes a receptor characterized by an unusual antibody reactivity being recognized by the anti‐KIR2DL1/S1 (EB6 and 11PB6) mAb and not by the anti‐KIR2DL3 (ECM‐41 and 180701) mAbs.^41^, ^49^ We evaluated whether the HP‐DM1/EB6 combination would allow distinguishing 2DL1^+^ from 2DL3*005^+^ NK cell fractions. To this aim, NK cells from 2DL3*005 ^+^ donors with *KIR A/A* genotype were stained with either HP‐DM1/EB6, HP‐DM1/HP‐MA4 or HP‐DM1/143211. Since HP‐MA4 does not recognize 2DL3*005 (Table 1),^45^ HP‐DM1/HP‐MA4 and HP‐DM1/EB6 dot plots were clearly different (the representative case TB520B is shown in Figure 2A). Thus, HP‐DM1/HP‐MA4, like HP‐DM1/143211 identified only 2DL1^+^ NK cells, while HP‐DM1/EB6 detected 2DL3*005^+^ cells, co‐expressing or not 2DL1. If we added an anti‐KIR2DL2/L3/S2 mAb (e.g., CH‐L or GL‐183) to HP‐DM1/EB6 staining, we found that all EB6^+^/HP‐DM1^−^ cells were also CH‐L^+^, displaying a diagonal staining pattern typical of co‐recognizing the same molecule (i.e., 2DL3*005) (Figure S3A, left panel). In the strategy described by Beziat et al.,^49^ based on a refined 15‐color flow cytometry panel and a flowchart with sequential quality controls (QCs), EB6/GL‐183 represented the first QC, and the diagonal staining pattern was the hallmark of the expressed 2DL3*005 allotype (Figure S3, middle panels).

Notably, the described mAb combinations can be very informative for 2DL3*005 ^+^ */2DS1* ^+^ donors. In the representative case TB344B shown in Figure 2B, 143211 and HP‐DM1 co‐stained 2DL1^+^ NK cells, HP‐MA4^+^/HP‐DM1^−^ identified 2DS1^+^ cells, while EB6^+^/HP‐DM1^−^ subset contained both 2DL3*005^+^ and 2DS1^+^ cells, as also documented by the additional staining with CH‐L mAb, detecting CH‐L^+^ (i.e., 2DL3*005^+^) and CH‐L^−^ (i.e., 2DS1^+^) subsets (Figure S3B, left panel). Finally, the simultaneous staining with HP‐DM1/EB6/HP‐MA4 mAb in the same panel could also be performed, providing a better identification of 2DL1^+^, 2DS1^+^, and 2DL3*005^+^ subsets (Figure 2B). Consistent data were obtained with the GL‐183/ECM‐41 mAb combination. In the 2DL3*005/*x donor TB520B, 2DL3*005^+^ NK cells were identified as the GL‐183^+^/ECM‐41^−^ subset (Figure S3A, right panel). In donor TB344B, carrying CenA/CenB (i.e., having only one *2DL3* allele), no ECM‐41 staining was observed (Figure S3B, right panel). Notably, the presence of 2DL3*005 allele in these donors was confirmed by SSP‐PCR analysis (Figure S2).

### 3.3 Use of HP‐DM1 mAb on NK cell phenotyping of telomeric *2DS5* ^+^ donors

**FIGURE 3:**
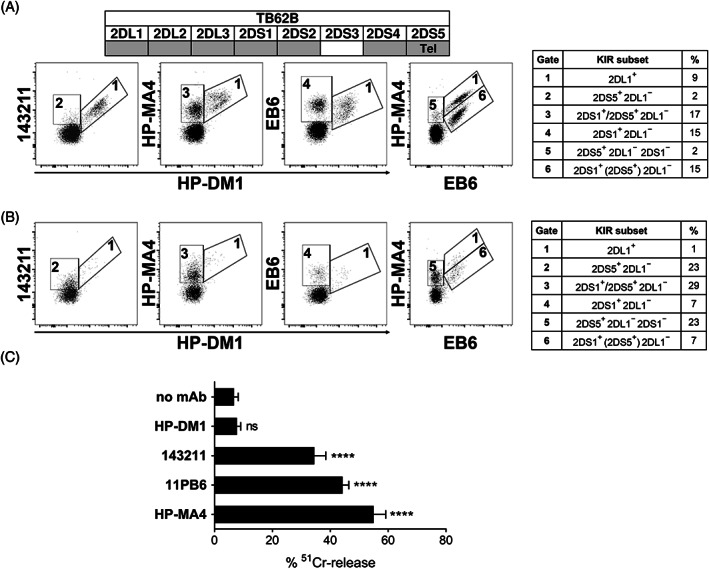
HP‐DM1 mAb uniquely recognizes 2DL1 in NK cells from donor carrying *2DS1* and telomeric *2DS5*. Immunofluorescence analyses were performed on resting NK cells derived from TB62B donor (A), characterized by the presence of telomeric *2DS5*, and the respective recipient at 6 months after the transplantation (B). The lineage III *KIR2D* gene profile is reported (positive and negative genes are identified as gray and white boxes, respectively). The whole *KIR* genotype and KIR‐L of TB62B donor and patient TB62 pre‐transplant are shown in Figure S1. HP‐DM1‐PE was used in combination with either 143211‐APC, HP‐MA4‐APC, or EB6‐APC; HP‐MA4‐APC/EB6‐PE staining is also shown. Different NK cell subsets (numbered gates) are identified by the indicated mAb combinations. KIR composition and percentage of NK cells included in each gate are shown in the table on the right side. In gate 3, cells express either 2DS1 or 2DS5 or both (the slash indicates these possibilities). (C) Polyclonal activated NK cells derived from the patient at 6 months after transplantation, shown in panel (B), were tested in R‐ADCC assay against ^51^Cr‐labeled P815, in the absence or presence of the indicated mAb. Data are shown as mean + SEM and are pooled from three independent experiments. E:T ratio was 4:1. ****p < 0.0001

In our cohort, mainly consisting of Italian individuals, *2DS5* was located primarily in Tel‐B region, in agreement with studies analyzing *KIR* polymorphism in European populations.^24^ In *2DL1* ^+^ */2DS1* ^+^ */2DS5* ^+^ donors, the combined staining of HP‐DM1 with 143211 mAb allowed the discrimination of 2DL1^+^ (HP‐DM1^+^/143211^+^) from 2DL1^−^/2DS5^+^ (HP‐DM1^−^/143211^+^) NK cell subsets (Figure 3A). In addition, with HP‐DM1/HP‐MA4 it was possible to discriminate the inhibitory 2DL1^+^ (HP‐DM1^+^/HP‐MA4^+^) NK cell subset from that expressing the activating 2DS1 and/or 2DS5 molecules (HP‐DM1^−^/HP‐MA4^+^). Staining performed using EB6 in combination with HP‐DM1 or HP‐MA4 allowed the visualization of additional NK cell subsets as detailed in Figure 3A. Apparently, EB6 did not recognize 2DS5 on NK cells since HP‐MA4^+^/EB6^−^ cells (i.e., gate 5) were detectable, as previously reported.^50^ Also in this case, the HP‐DM1/EB6/HP‐MA4 mAb combination was useful to identify the 2DL1^+^ subset, providing more precise discrimination between 2DS1^+^ (recognized by both EB6 and HP‐MA4 mAb) and 2DS5^+^ (stained by HP‐MA4 mAb) subsets (Figure S4A). In this donor, as in most studied cases, the frequency of the 2DS5^+^ NK cell subset was low (~2%), at least among 2DL1^−^ cells. *KIR* genotype and KIR‐L of the donor (TB62B) and the related patient before transplantation (TB62) are shown in Figure S1 panel A and B, respectively. Interestingly, testing the NK cell phenotype of the reconstituted repertoire in the leukemia patient following αβT/B‐depleted haplo‐HSCT from this donor, we observed a marked expansion of the donor‐derived 2DS5^+^ subset (from 2% to 23%) and a reduction of 2DL1^+^ NK cells (from 9% to 1%) (Figure 3B). In R‐ADCC assay, all the mentioned mAb except HP‐DM1 triggered lysis of activated NK cells, consistent with the engagement of 2DS5 by 143211, 2DS1 by EB6, and both 2DS1 and 2DS5 by HP‐MA4 mAb (Figure 3C).

### 3.4 Impact of HP‐DM1 mAb on NK cell phenotype of centromeric *2DS5* ^+^ donors

**FIGURE 4:**
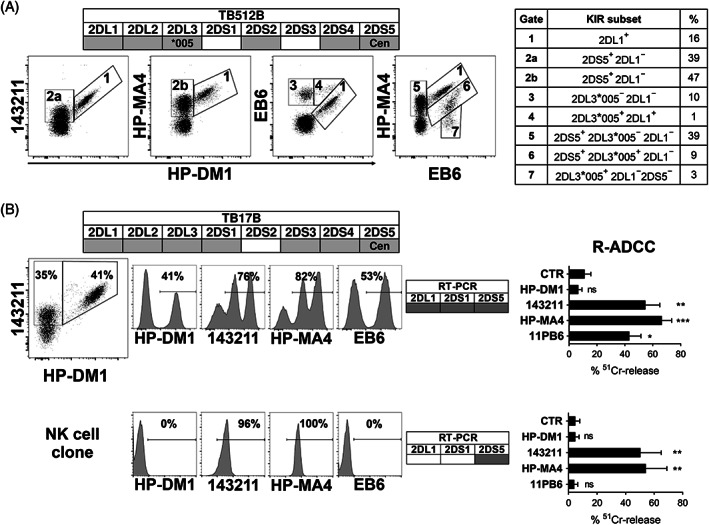
HP‐DM1 mAb allows to dissect 2DL1^+^ from 2DS5^+^ NK cells in donors carrying centromeric *2DS5*. (A and B) Immunofluorescence, transcript and functional analyses performed using NK cells derived from two donors (TB512B, and TB17B), characterized by centromeric *2DS5*. The lineage III *KIR2D* gene profiles are shown (positive and negative genes are reported as gray and while boxes, respectively), while the whole *KIR* genotype and KIR‐L are reported in Figure S1. (A) Resting NK cells derived from donor TB512B were stained with HP‐DM1‐PE in combination with 143211‐APC, HP‐MA4‐APC, or EB6‐APC; HP‐MA4‐APC/EB6‐PE staining is also shown. Different NK cell subsets are identified by numbered gates. Definition of KIR subsets and percentage of NK cells included in each gate are reported in the table on the right side. (B) Activated polyclonal (upper panels) or clonal (lower panels) NK cells derived from donor TB17B were analyzed by immunofluorescence. Percentages of positive cells, RT‐PCR of the indicated KIR2D transcript (the positive transcripts are identified as gray, while negative as white boxes), and R‐ADCC against P815 in the absence or presence of the indicated mAb are reported. ****p < 0.0001; ***p < 0.001; **p < 0.01

We had the opportunity to also analyze donors from Africa and Central America, finding two characterized by a centromeric *2DS5* allele; both donors have C1, C2, and Bw4 KIR‐L (Figure S1). While the presence of a *2DS5* centromeric allele in donor TB512B was quite evident (this donor is *2DL5B* ^+^, *2DL5A* ^−^, *2DS3* ^−^, and *2DS5* ^+^, Figure S1), in donor TB17B (*2DL5B* ^+^, *2DL5A* ^+^, *2DS3* ^+^, and *2DS5* ^+^, Figure S1) the identification of the *2DS5* allele was required. To this end we sequenced *2DS5* transcripts from polyclonal activated NK cells. This analysis revealed the presence of 2DS5*006, an allele that has been described only in the centromeric region.^29^ Notably, both individuals were characterized by exceptionally high percentages of 2DS5^+^ NK cells. Indeed, in the *2DL1* ^+^ */2DS1* ^−^ */2DS5* ^+^ TB512B donor (of Ugandan origin), the 2DS5^+^/2DL1^−^ subset was quantified as 35% or 46% using 143211/HP‐DM1 or HP‐MA4/HP‐DM1 combinations, respectively, showing a better 2DS5 staining by HP‐MA4 than 143211 mAb (Figure 4A). This donor was also characterized by the presence of 2DL3*005 (Figure S2), leading to a further complication in KIR subset definition. Thus, the presence of 2DL3*005^+^ subset in TB512B NK cells was detected by the bright staining of EB6, partially co‐expressing 2DL1 (in EB6/HP‐DM1) and/or 2DS5 (in EB6/HP‐MA4) (Figure 4A). As expected, EB6^+^/HP‐DM1^−^ cells were also CH‐L^+^, and the staining with GL‐183/ECM‐41 mAb did not show any ECM41^+^ cells (Figure S3C). The HP‐DM1/EB6/HP‐MA4 mAb combination further defined various KIR^+^ subsets (Figure S4B). In addition to the evidence that 2DS5^+^ 2DL1^−^ NK cells could co‐express 2DL3*005 (gate 6 in Figure 4A), we also found that they co‐expressed 2DL2, 3DL1 and/or NKG2A, supporting the notion of their licensing state (Figure S5A).

A highly represented 2DS5^+^ NK cell subset was also detected in the polyclonal population derived from the *2DL1* ^+^ */2DS1* ^+^ */2DS5* ^+^ TB17B donor (of Cuban origin) (Figure 4B). Notably, the profiles obtained using 143211 and HP‐MA4 revealed both dull and bright staining, with the weak reactivity suggesting 2DS5 recognition. Again, R‐ADCC assay provided a functional demonstration that only HP‐DM1 mAb was reacting exclusively with the inhibitory receptor 2DL1, while 143211 (also recognizing 2DS5), 11PB6 mAb (also recognizing 2DS1), and HP‐MA4 (also recognizing 2DS1 and 2DS5) triggered lysis. These findings were further documented at the clonal level. We selected HP‐MA4^+^/143211^+^/EB6^−^/HP‐DM1^−^ (with dull staining of HP‐MA4 and 143211) NK cell clones, in which HP‐MA4 and 143211 mAb triggered lysis in R‐ADCC, revealing the activating function of the engaged receptor (i.e., 2DS5). By RT‐PCR, we detected the presence of *2DS5* transcript and the absence of *2DL1* and *2DS1* (a representative clone is shown in Figure 4B). These 2DS5^+^ 2DL1^−^ NK cell clones appeared to be educated either through the expression of self‐reactive iKIRs as 2DL2/L3 and 3DL1, or through NKG2A (Figure S5B).

### 3.5 HP‐DM1 recognizes the 2DL1 epitope relevant for binding to HLA‐C

**FIGURE 5:**
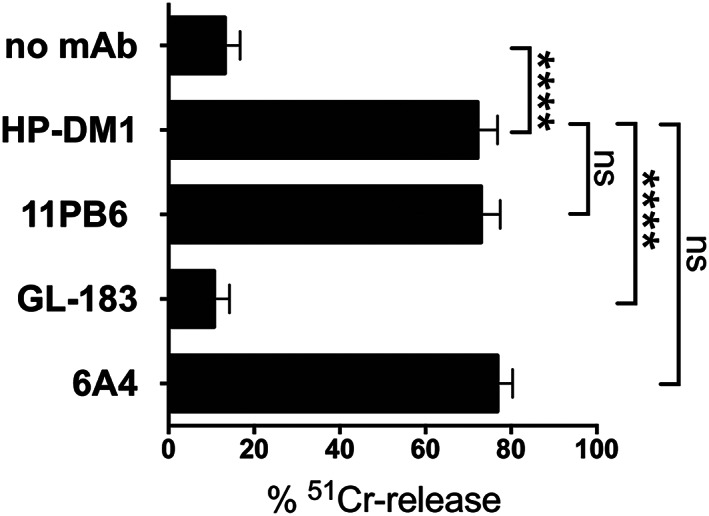
HP‐DM1 mAb blocks 2DL1/HLA‐C2 interaction. Polyclonal activated NK cells with a large 2DL1^+^ cell subset were derived from two donors (TB372B and D9, whose *KIR* genotypes and KIR‐L are reported in Figure S1), and tested in ^51^Cr‐release assays against C1R B‐EBV cell line (HLA‐C2) in the absence or in the presence of the indicated mAb. E:T ratio was 5:1. Data are shown as mean + SEM and are pooled from duplicates in two independent experiments. ****p < 0.0001.

Epitope mapping of HP‐DM1 suggested that this mAb binds to residues involved in ligand recognition.^45^ To verify this hypothesis, we analyzed whether pre‐treatment of NK cells with HP‐DM1 mAb could mask the interaction between 2DL1 and HLA‐C allotypes with C2 epitope. To this end, we tested in cytolytic assays activated NK cells derived from two donors (TB372B and D9) characterized by an expansion of 2DL1^+^ subset (82% and 98%, respectively) against C1R B‐EBV cell line, expressing HLA‐C*04:01 (C2 epitope) (Figure 5). These target cells were resistant to lysis, and, upon 2DL1 masking by HP‐DM1 mAb, the killing could be efficiently restored at the same level detected using 11PB6 mAb and by blocking HLA class I molecules using 6A4 mAb.^51^ The anti‐2DL2/L3/S2 GL‐183 mAb was used as a negative control. These data demonstrated that HP‐DM1 mAb blocks the HLA‐C binding site of 2DL1.

## 4 DISCUSSION

The high diversity of NK cell phenotypic repertoire may depend on different factors including *KIR* gene content, allelic polymorphism, transcriptional regulation, copy number variation, self *HLA* class I polymorphism, and cytomegalovirus (CMV) infection.^36^, ^52^, ^53^, ^54^, ^55^, ^56^, ^57^, ^58^, ^59^ Hence, in addition to *KIR* and *HLA* class I genotyping, phenotypic characterization of KIR repertoire becomes an important tool for basic and clinical studies. In this context, given the frequent reactivity of mAbs with different KIRs, the combined use of well‐characterized reagents is required to correctly identify certain KIR expressing subsets. To this end, knowledge of the residues relevant in their reactivity is helpful. Regarding anti‐KIR2DL1 mAbs, epitope mapping was previously reported for EB6 mAb (E35 and R50),^41^ being as well achieved for HP‐DM1 (M44, S67, R68, and T70) and 143211 (R68 and T70) mAb.^45^ Definition of KIR residues relevant for HP‐DM1 binding and analysis of *KIR* sequences allowed to predict its exclusive reactivity with 2DL1, covering all allotypes so far identified excepting 2DL1*022 (characterized by K44) and, probably, 2DL1*020 (carrying G67), described in sub‐Saharan African populations which were scarcely represented in our cohort. In European populations, the most common *KIR2DL1* alleles on the A haplotype are KIR2DL1*003, *002 or *001, while the dominant allele on B haplotype is KIR2DL1*004.^15^, ^57^ Regarding 2DL1*022, this allele has been described in KhoeSan population of Southern Africa, that are characterized by a unique demographic history and are genetically diverse from other human populations.^16^, ^60^ Another potential exception is the predicted HP‐DM1 binding to 2DS1*013, which carries M44, S67, R68, and T70, like most 2DL1 and different, at position 70, from the other 2DS1 allotypes. To the best of our knowledge, no information is available about the frequency of 2DS1*013. Altogether these exceptions should not be relevant when analyzing 2DL1 expression by HP‐DM1 mAb in European populations.

On that basis, HP‐DM1 reactivity with NK cells derived from potential HSCT donors with a known *KIR* gene profile was assessed, employing different flow cytometry staining strategies to define the proportions and functional activity of distinct KIR^+^ subsets. Technically, whenever combinations of mAbs react with different epitopes of the same KIR, the staining procedure should be carefully assessed to prevent binding interferences. In our experience, when EB6 and HP‐MA4 mAbs were combined, a sequential staining was required to preserve the optimal reactivity of both mAbs, with a procedure (see Methods) different from that described by Czaja et al.^50^ In the other mAb combinations including HP‐DM1, simultaneous incubation with different reagents was acceptable. Notably, only HP‐DM1 mAb precisely identified 2DL1^+^ NK cells, and its use in combination with other mAb allowed the identification of distinct subsets co‐expressing or not 2DL3*005, 2DS1, and/or 2DS5. Moreover, this approach contributed to clarify potentially ambiguous staining patterns. For instance, the use of HP‐DM1 enabled to precisely define the KIR2DL1^+^ subset in the presence of 2DL3*005, which is recognized by anti‐KIR2DL1/S1 EB6 and 11PB6 mAbs. Adding HP‐MA4 mAb to these two mAb allowed to dissect 2DL1^+^, 2DS1^+^, and 2DL3*005^+^ subsets. In the same line, as predicted by detailed epitope mapping,^45^ the combination of anti‐KIR mAb employed in this study would allow the discrimination of canonical C2‐specific 2DL1 allotypes (HP‐DM1^+^) from the atypical C1‐specific 2DL1*022 (HP‐DM1^−^ but 143211^+^, EB6^+^, and HP‐MA4^+^). In the case of TB512B donor from Uganda, characterized by centromeric *2DS5* and 2DL3*005 (Figure 4A), the presence of 2DL1*022 could be excluded because EB6^+^/HP‐DM1^−^ cells were all CH‐L^+^ and thus 2DL3*005^+^.

HP‐DM1 mAb appears of particular interest for immunophenotyping *2DS5* ^+^ donors, because the other available anti‐KIR2DL1 143211 mAb does also bind 2DS5. Thus, HP‐DM1/143211 co‐staining allows the distinction of 2DL1 from 2DS5, which is also efficiently recognized by HP‐MA4.^50^ On that basis, the HP‐DM1/HP‐MA4/EB6 mAb combination was set up to discriminate 2DL1, 2DS5, 2DS1, and 2DL3*005. Remarkably, through this approach we detected higher proportions of 2DS5^+^ NK cells in centromeric than telomeric *2DS5* ^+^ individuals, to be confirmed by studying a larger cohort. These data are consistent with the expression levels detected on the surface of NKL cells transfected with different *2DS5* alleles, showing lower levels of 2DS5*002 compared with the other allotypes.^61^ This depends on polymorphic variation that impacts the N‐linked glycosylation, maturation and/or transport of the protein to the membrane.^61^ Moreover, the use of this mAb panel for monitoring the NK cell reconstitution after HSCT allowed us to observe in a patient a marked expansion of the 2DS5^+^ subset, with a concomitant contraction of the 2DL1^+^ population. As compared with 2DS5^+^ NK cells (2%) detected in the donor carrying telomeric *2DS5* (Figure 3), their proportions raised up to 23% at 6 and 9 months post‐HSCT, declining at 1 year (5%). It should be noted that donor but not patient *KIR* genotype contained *2DS5* (Figure S1A,B), further sustaining that NK cells analyzed in the patient after transplantation were donor‐derived. It is uncertain which factor(s) promoted these phenotypic changes, and their putative relation with CMV reactivation or GvHD, that occurred in this patient, deserves further investigation. The potential influence of KIR‐L in the donor (C1 and Bw4) and in the patient (C1, C2, and Bw4) remains also undefined, because the telomeric 2DS5*002, the predominant allotype in Europeans, does not bind HLA‐A, ‐B or –C.^29^ Actually, the more accurate definition of the 2DS5^+^ subset achieved with our staining protocol paves the way for phenotypic and functional studies which might contribute to better understanding the biological role of this aKIR.^22^, ^62^

Altogether our study shows that HP‐DM1 mAb is a unique reagent that specifically recognizes 2DL1 molecules, becoming valuable to more precisely characterize the KIR repertoire by multicolor flow or mass cytometry. In the context of haploidentical HSCT, where KIR‐HLA class I mismatch in graft versus host direction has been associated to NK‐mediated graft versus leukemia effect,^14^, ^40^, ^63^, ^64^ the frequencies of 2DL1^+^ alloreactive NK cells might be precisely monitored by multicolour flow cytometry, combining HP‐DM1 with the other iKIR‐specific mAbs. Importantly, HP‐DM1 mAb reacts with a conformational epitope including positions M44, R68, and T70 that have been described to be involved in the HLA‐C binding site of 2DL1.^15^, ^65^ Indeed, we provide functional evidence supporting that HP‐DM1 mAb‐mediated masking of 2DL1 efficiently blocked the recognition of HLA‐C molecules carrying C2 epitope, with the potential advantage with respect to other mAb as 11PB6 to avoid concomitant blocking of the activating 2DS1/HLA‐C2,^14^ and the inhibitory 2DL3*005/HLA‐C1 interaction.^41^ On that basis, engineering therapeutic mAbs with antagonistic effects based on HP‐DM1 structure might be of potential translational relevance. An example can be represented by selectively blocking donor 2DL1 interaction with recipient carrying C2^+^ HLA‐C in haplo‐HSCT to possibly enhance NK alloreactivity, particularly in certain settings (e.g., donor C1/C2 and recipient C2/C2). A caveat would be to prevent potential interferences with 2DL1‐dependent “education” following donor NK cell differentiation, thus requiring to define the suitable schedule for administration of the antagonistic mAb. In this regard, it was reported that treatment of cancer patient by infusing IPH2101 (the fully human antibody specific for both inhibitory 2DL1/L2/L3 and activating 2DS1/S2) had limited side effects,^66^ but also reduced anti‐tumor activity.^67^ Indeed, loss of free KIR2D surface molecules by trogocytosis correlated with reduction of NK‐cell function, presumably interfering with licensing.^67^ In the haplo‐HSCT setting described above, it is conceivable that selectively blocking 2DL1 instead of all KIR2D molecules might prevent dampening of NK cell activity, given the preserved expression of 2DL2/L3, recognizing donor but not recipient cells. In addition, the presence and activity of 2DS1/S2 would be maintained on the NK cell surface.

## AUTHOR CONTRIBUTIONS

Raffaella Meazza, Michela Falco, and Daniela Pende designed the study, analyzed and interpreted the data. Miguel Lopez‐Botet generated and selected HP‐DM1 mAb, proposing its collaborative characterization. Raffaella Meazza, Paolo Canevali, Fabrizio Loiacono, Natalia Colomar‐Carando, Aura Muntasell, and Anna Rea performed phenotypic and functional analyses. Michela Falco performed *KIR* genotype and analyzed *KIR* alleles. Raffaella Meazza, Paolo Canevali, Fabrizio Loiacono obtained polyclonal and clonal NK cells. Franco Locatelli coordinated the study of haplo‐HSCT donors and patients. Raffaella Meazza, Michela Falco, Miguel Lopez‐Botet, and Daniela Pende wrote the manuscript. Aura Muntasell, Maria Cristina Mingari, and Lorenzo Moretta critically revised the manuscript. Michela Falco, Lorenzo Moretta, Franco Locatelli, Miguel Lopez‐Botet, and Daniela Pende provided fundings. All the authors approved the final version.

## CONFLICT OF INTEREST

Miguel López‐Botet declares that HP‐DM1 and HP‐MA4 are commercially available through license agreements signed by Universitat Pompeu Fabra. The other authors declare no conflict of interest.

## Supporting information


**Appendix S1** Supporting Information

## Data Availability

The data that support the findings of this study are available from the corresponding author upon reasonable request.
