# Supplementary material for: Characterization of KIR + NK cell subsets with a monoclonal antibody selectively recognizing KIR2DL1 and blocking the specific interaction with HLA‐C
Source: HLA. 2022 May 15;100(2):119–32. doi: 10.1111/tan.14640 (PMC9543057; doi:10.1111/tan.14640)
Supplement: Supplementary file 1 — Appendix S1 Supporting Information [file TAN-100-119-s001.docx]

**Supplementary Material**

**Manuscript ID HLA-Jan-2022-2218-OA entitled “Characterization of KIR^+^ NK cell subsets with a monoclonal antibody selectively recognizing KIR2DL1 and blocking the specific interaction with HLA-C” by R. Meazza, M. Falco et al.**

**
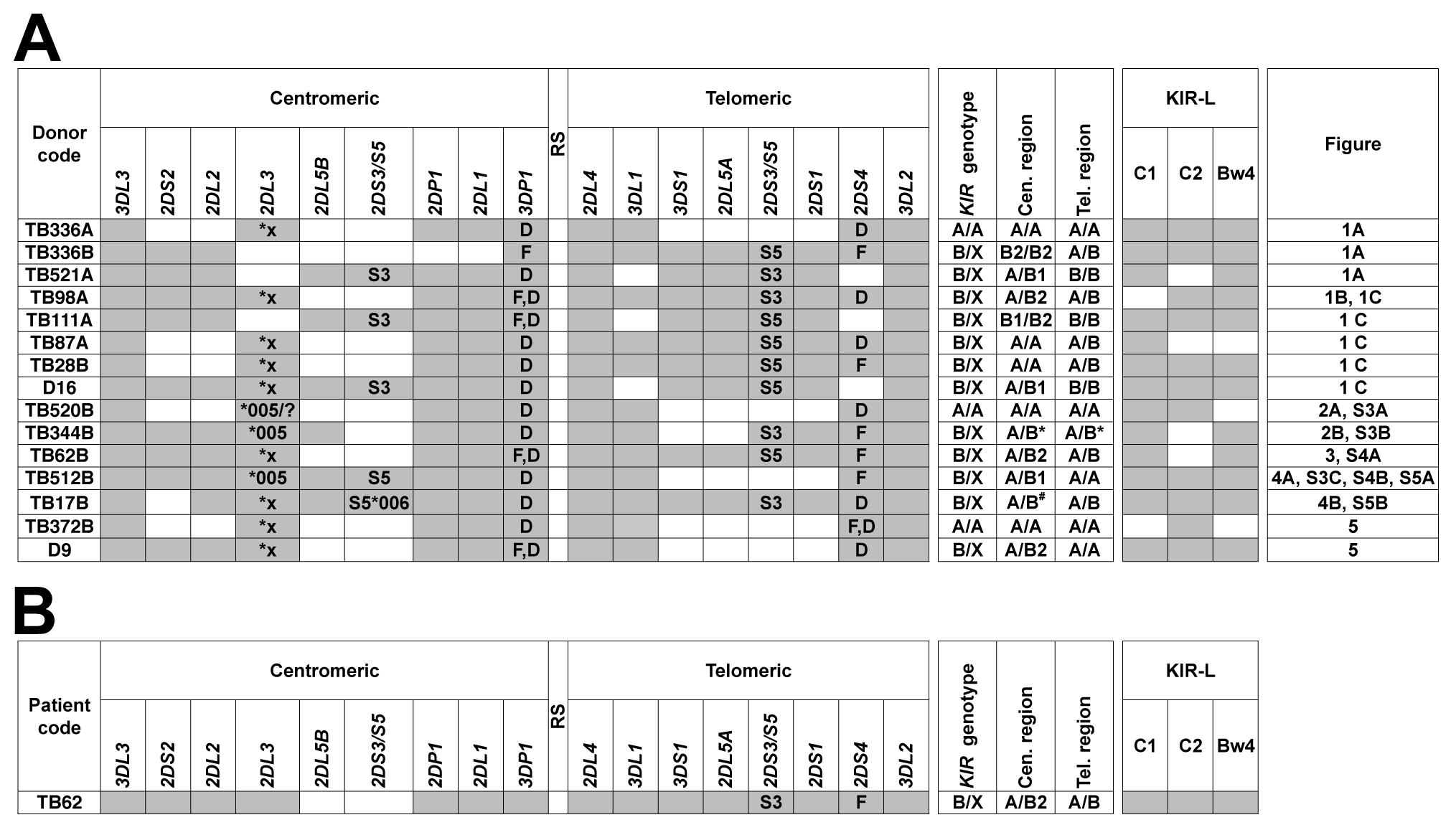
**

**Figure S1**: ***KIR* gene profiles and KIR ligands of the individuals included in the study. (A)** Fifteen donors of haplo-HSCT and **(B)** one patient (TB62) before being transplanted from TB62B were analyzed. The *KIR* gene presence or absence is indicated with grey or white boxes, respectively. In 2DL3 boxes, *005 indicates the presence of a *2DL3*005* allele, *005/? of at least one *2DL3*005* allele, while *x all the other alleles. S3 and S5 are used to identify *2DS3* and *2DS5*, respectively. The presence of *3DP1* alleles including or not exon 2, and *2DS4* alleles coding for surface or truncated receptor are reported as F and D, respectively. B* indicates that donor TB344B carries a contracted haplotype missing the seven *KIR* genes included between *2DL5B* and *2DS3*. B^#^ indicates that the donor TB17B is characterized by a B1 centromeric region missing *2DS2*. The gene order is based on *KIR* haplotype published sequences. For each individual, the presence or absence of C1, C2, and Bw4 KIR ligands (KIR-L) are also indicated with grey and white boxes, respectively.


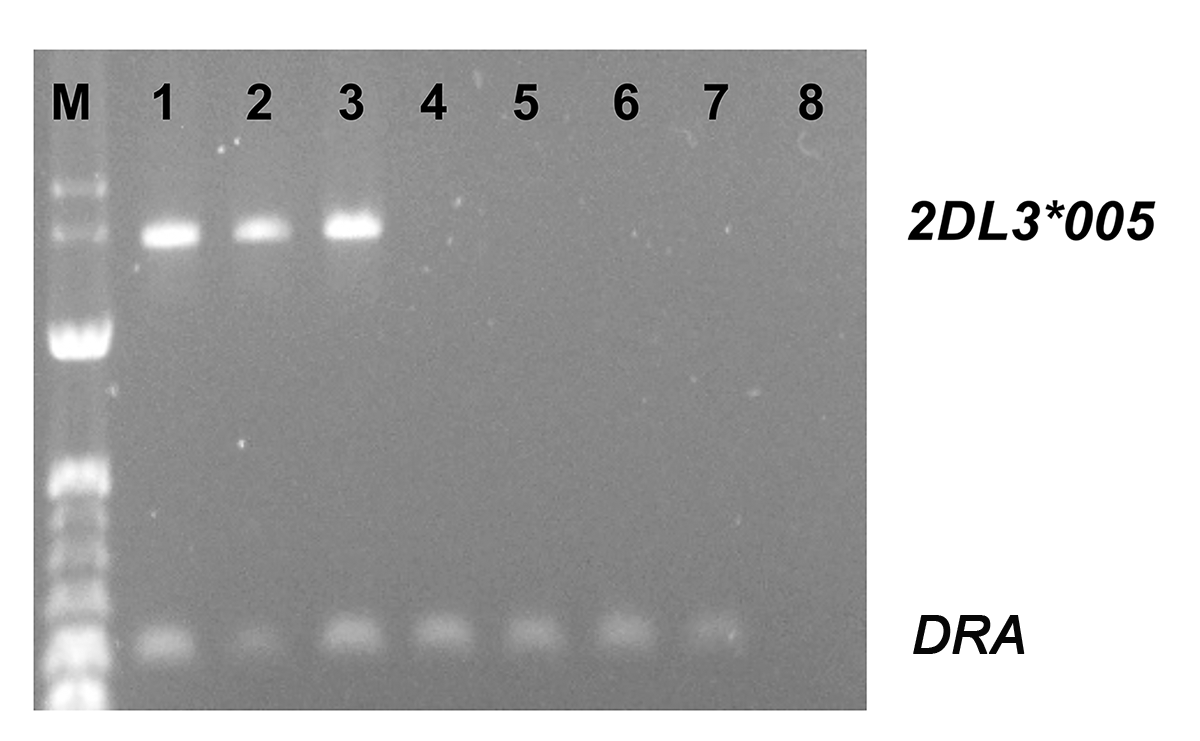


**Figure S2: Detection of *2DL3*005* allele.** SSP-PCR analysis of seven *2DL3*^+^ donors three of which are characterized by *2DL3*005* (lanes 1-3: TB520B, TB344B, and TB512B) and 4 representative donors carrying different *2DL3* alleles (lanes 4-7: TB336B, TB98A, TB87A, and TB28B). Negative control was included in the test (line 8). M indicates the m.w. marker. PCR of an *HLA-DRA* gene fragment was used as internal control.


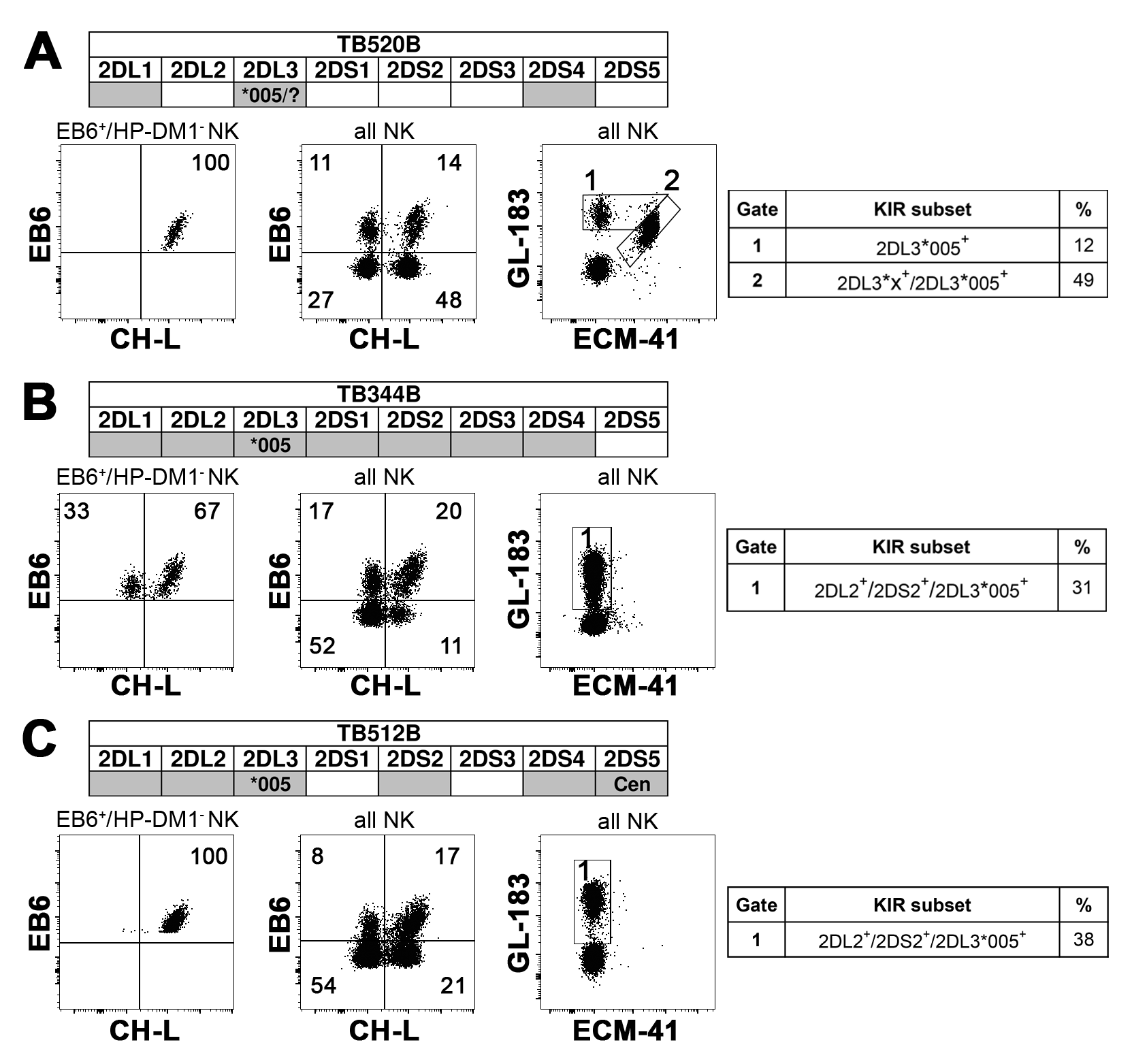


**Figure S3: Phenotypic definition of 2DL3*005 allotype.** Immunofluorescence and flow cytofluorimetric analysis of resting NK cells (gating on CD3^–^CD56^+^ cells of PBMC) from one *2DL3*005/?* donor **(A)** and two *2DL2/2DS2/2DL3*005* donors **(B, C)** were performed to define the presence of 2DL3*005 allotype. **Left panels:** anti-KIR mAbs (EB6-APC, HP-DM1-PE, CH-L-FITC) were used in combination with anti-CD3-BV510 and anti-CD56-BV421. Gating on EB6^+^/HP-DM1^−^ NK cell subset, EB6^+^/CHL^+^ cells display a diagonal staining pattern, suggesting the co-staining of the same molecule (i.e., 2DL3*005); in *2DS1^−^* TB520B and TB512B donors, all EB6^+^ cells are CH-L^+^, while in *2DS1^+^* TB344B donor the EB6^+^/CH-L^−^ subset represents 2DS1^+^ cells. Numbers indicate the % of positive cells. **Middle panels:** anti-KIR mAbs (EB6-APC, HP-DM1-PE, CH-L-FITC) were used in combination with anti-CD3-BV510 and anti-CD56-BV421. Gating on all NK cells, EB6^+^/CHL^+^ cells display a diagonal staining pattern. **Right panels:** ECM-41 mAb, followed by anti-IgM-FITC second reagent, was used in combination with GL-183-PE mAb and anti-CD3-BV510 and anti-CD56-BV421. Different NK cell subsets can be identified by gates, which are numbered and defined for their KIR composition and percentage of NK cells included (table on the right side). In gates, the slash indicates that cells can express either one or another KIR or all together.


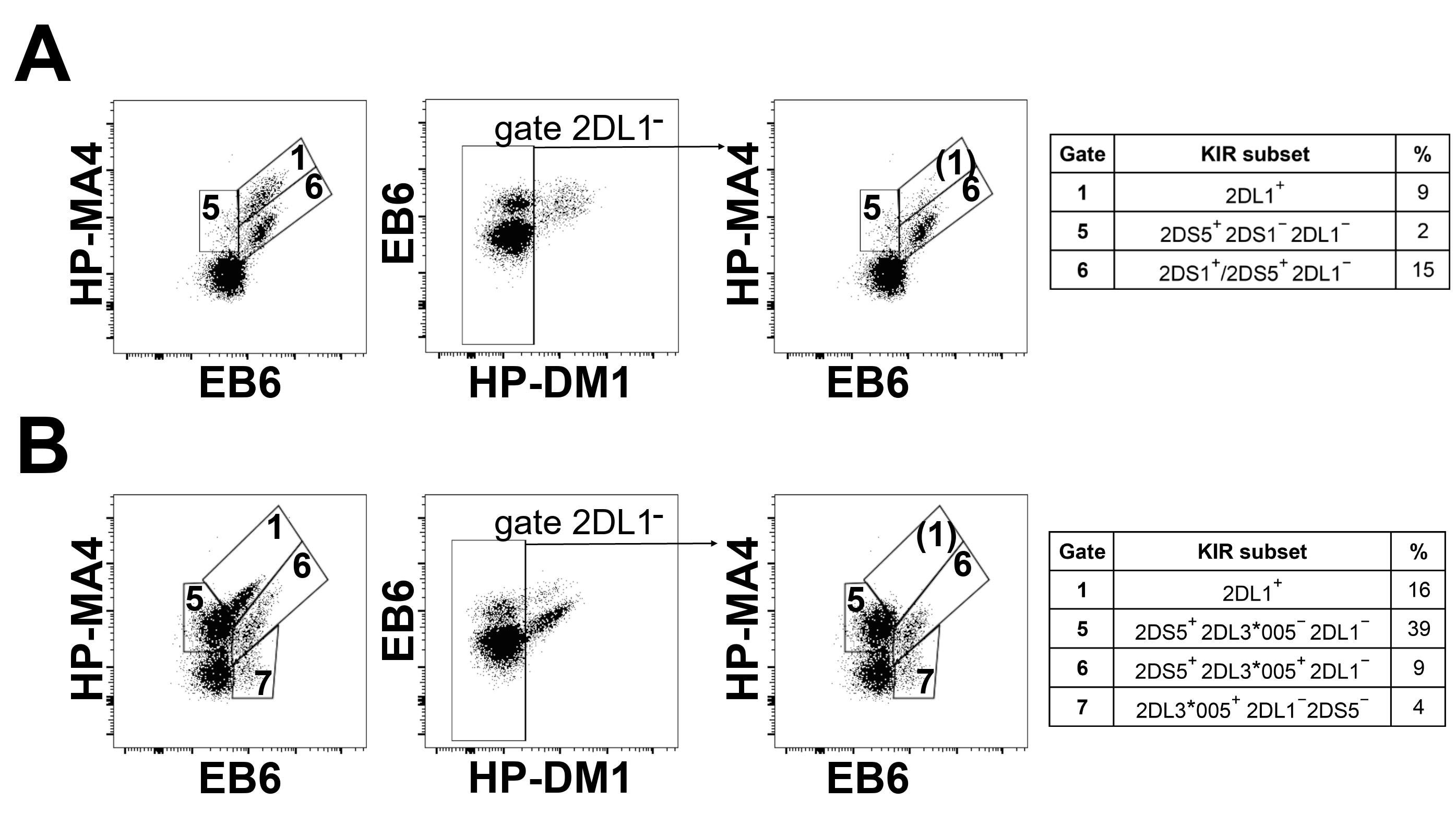


**Figure S4: Cytofluorimetric analysis using HP-DM1/EB6/HP-MA4 mAb combination.** Immunofluorescence analysis using HP-DM1-PE/EB6-PC7/HP-MA4-APC was performed on NK cells (gating on CD3^–^ CD56^+^ cells of PBMC) derived from TB62B donor **(A)** (also shown in Figure 3), and TB512B donor **(B)** (also shown in Figure 4), respectively. The *KIR* gene profile of these donors is shown in Figure S1. Different NK cell subsets can be identified by gates, which are numbered and defined for their KIR composition and percentage of NK cells included (table on the right side). When gating on HP-DM1*^–^* (i.e., 2DL1*^–^*) cells, gate N° 1 remains with very few cells and so it is indicated within brackets. In gates, the slash indicates that cells can express either one or another KIR or all together.

**
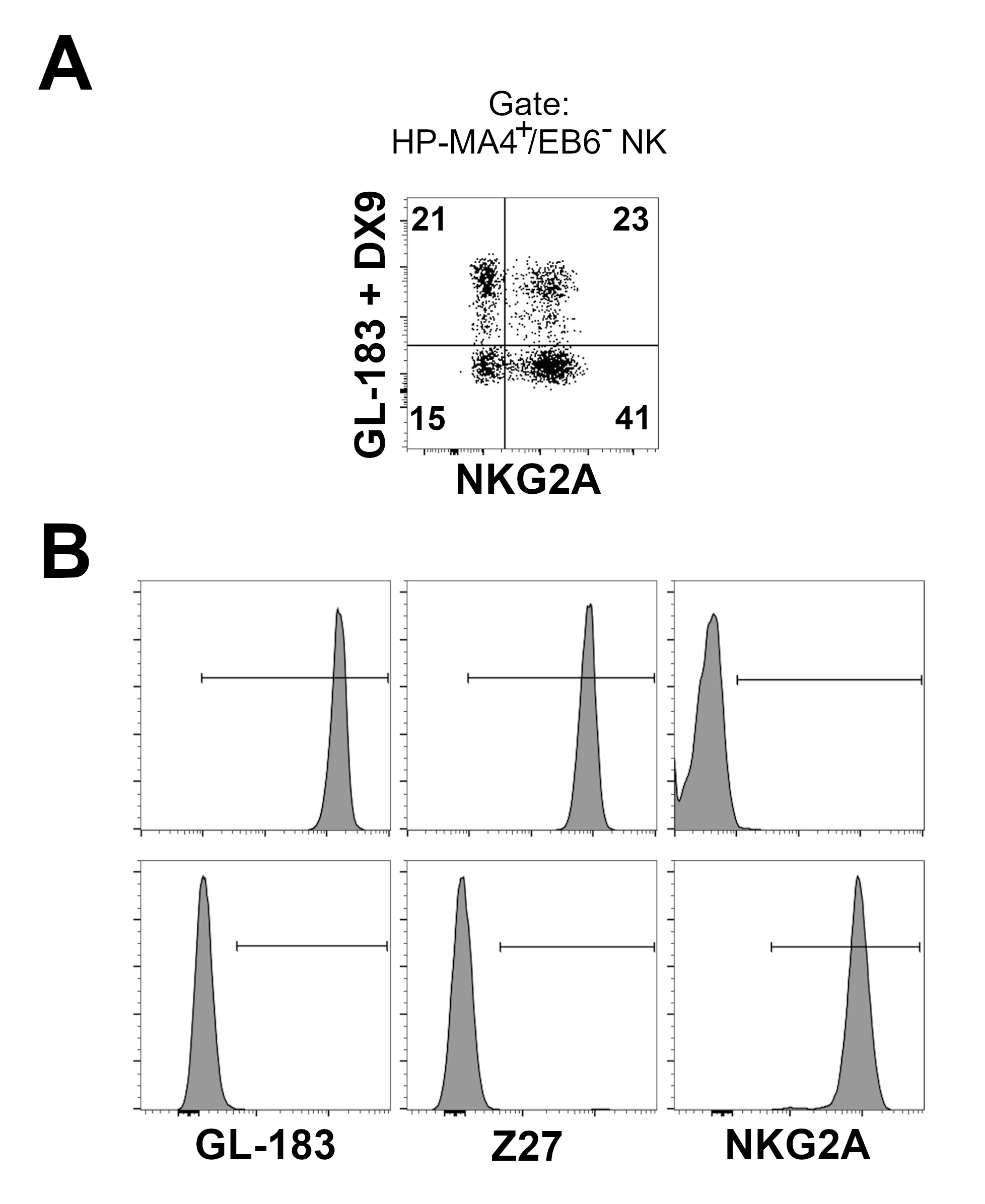
**

**Figure S5: Cytofluorimetric analysis of 2DL1*^–^* NK cells expressing centromeric 2DS5. (A)** Resting NK cells (CD3^–^ CD56^+^ PBMC) from TB512B were analyzed staining with HP-MA4-APC, EB6-PE, GL-183-PC7, DX9-PE-Vio770, and NKG2A-FITC mAb combination. After gating on HP-MA4^+^ EB6^–^ cells (i.e., 2DS5^+^ 2DL3*005^–^ 2DL1^–^, see Figure 4A), the expression of 2DL2/S2 together with 3DL1 versus NKG2A was evaluated. **(B)** Two representative 2DL1^–^ 2DS1^–^ 2DS5^+^ NK cell clones (upper clone is that shown in Figure 4B) derived from TB17B were analyzed for expression of 2DL2/L3, 3DL1/S1, and NKG2A.
